# The Association Between Deficiency of Vitamin D and Diabetes Mellitus Type 2 (DMT2)

**DOI:** 10.7759/cureus.22221

**Published:** 2022-02-14

**Authors:** Muhammad Khudayar, Ammar Nadeem, Maham N Lodi, Kubra Rehman, Syed I Jawaid, Ayesha Mehboob, Abdul S Aleem, Rida E Fatima Mirza, Moiz Ahmed, Kiran Abbas

**Affiliations:** 1 Department of Medicine, Shifa International Hospital, Islamabad, PAK; 2 Department of Medicine, Jinnah Sindh Medical University, Karachi, PAK; 3 Department of Medicine, Shaheed Mohtarma Benazir Bhutto Medical College, Karachi, PAK; 4 Department of Medicine, Liaquat College of Medicine and Dentistry, Karachi, PAK; 5 Department of Medicine, United Medical and Dental College, Karachi, PAK; 6 Department of Emergency Medicine, Royal Infirmary of Edinburgh, Edinburgh, GBR; 7 Department of Medicine, Jinnah Postgraduate Medical Centre, Karachi, PAK; 8 Medicine and Surgery, Sindh Medical College, Karachi, PAK

**Keywords:** 25- dihydroxy vitamin d, vitamin d levels (vit d level), 25 (oh) vitamin d, types 2 diabetes, patient

## Abstract

Background

The impact of vitamin D deficiency on the incidence of various diseases and its relationship with the progression of diabetes mellitus type 2 (DMT2) is still controversial. The present study evaluated the incidence of vitamin D deficiency in patients with DMT2.

Methodology

A cross-sectional study was conducted in a tertiary care hospital, Sindh, Pakistan from October 2020 to September 2021. A total of 525 patients of DMT2 were recruited. Another 525 patients acted as healthy controls. In patients with DMT2, blood samples were taken in the morning to measure vitamin D levels. All socio-demographic and clinical data were documented in a predefined pro forma. The association between the incidence of DMT2 and hypovitaminosis was explored.

Results

The mean age of the patients was 50 ± 5.5 years. There were 100 (54.1%) male and 85 (45.9%) female patients. The mean duration of diabetes of the patients was 6.8 ± 2.4 years. The mean serum 25-hydroxy vitamin D level was 22.3 ±10.4 ng/ml. In the case group, the majority of the patients had vitamin D deficiency i.e. 54.1%, while only 25.9% of controls had hypovitaminosis. Vitamin D deficiency was significantly correlated with the occurrence of DMT2 (p<0.0001).

Conclusion

The current study indicates that patients with diabetes mellitus type 2 (DMT2) more frequently suffer from vitamin D deficiency. Those patients with vitamin D deficiency and DMT2 can benefit from vitamin D replenishment. This may help improve glycemic control in these patients. This study served as a catalyst for future studies where the relationship between hypovitaminosis and insulin resistance can be thoroughly explored.

## Introduction

The incidence of Diabetes Mellitus type 2 (DMT2) is increasing at an alarming rate both nationally and internationally [1]. Recent evidence suggests that vitamin D and calcium homeostasis may also be important for a variety of non-skeletal outcomes including neuromuscular function, psoriasis, multiple sclerosis, colorectal, and prostate cancer [2,3]. Less well-known is the impact of vitamin D deficiency on depression, immunity and autoimmunity, obesity, and the progression of DMT2 [4]. Based on basic and animal studies, vitamin D has also been suspected as a modifier for diabetes risk [5]. 

Individuals with vitamin D deficiency exhibited a 48 percent reduction in insulin secretion compared to individuals with optimal levels of vitamin D. Thus, indicating that vitamin D stimulates the pancreas to produce insulin [6]. Beta-cell dysfunction and insulin resistance are also associated with low vitamin D levels and substantial improvement in hemoglobin A1c (HbA1c) levels with the reversal of vitamin D deficiency. Vitamin D deficiency increased insulin resistance, decreased insulin production, and was associated with metabolic syndrome [7]. 

Vitamin D replenishment improves glycemia and insulin secretion in patients with DMT2 with established hypovitaminosis D, thereby suggesting a role for vitamin D in the pathogenesis of DMT2 [8]. In a study, it was found that the majority of the DMT2 individuals had hypovitaminosis, compared with 36% of the individuals with type 1 diabetes. Thereby, indicating that vitamin D deficiency is more common in DMT2 than in type 1 diabetes mellitus [9]. Recent evidence has demonstrated that those with DMT2 who have hypovitaminosis D are more likely to have increased C-reactive protein (an indicator of inflammation), fibrinogen, and high levels of A1c compared with other diabetic patients [10].

Diabetes is a major health problem and is associated with high morbidity and mortality in the form of chronic renal failure and ischemic heart disease. Vitamin D deficiency is a potentially correctable cause of diabetes. The 25-hydroxy vitamin D test is the most accurate measure of the amount of vitamin D in the body. The present study aimed at determining the frequency of vitamin D deficiency in patients with DMT2. This could help establish the role of interventions to correct vitamin D deficiency in individuals with diabetes thus subsiding complications and improving patient outcomes.

## Materials and methods

A case-control study was conducted at a tertiary care center, Sindh, Pakistan between October 2020 to September 2021. A non-probability purposive sampling technique was used to enroll the participants. The acquisition of data started after ethical approval was obtained from the Institutional Review Board (IRB) of Jinnah Postgraduate Medical Center (approval number: F2-65/2020-GENL/5324/JPMC). 

All patients between the ages of 30 and 60 years, irrespective of gender, diagnosed with diabetes mellitus type 2 (DMT2) were included in our study. Diagnosis of DMT2 was considered when the fasting blood glucose levels were greater than 110 mg/dl and two-hour postprandial blood glucose was higher than 140 mg/dl. Patients who had significantly high serum urea and serum creatinine, those who were on calcium or vitamin D supplements, or those with malabsorption, and significant liver disease were not eligible to partake in the study. Informed verbal and written consent was procured from all patients and the control group prior to the study.

A total of 525 patients with DMT2 were recruited and labeled as cases. Individuals visiting the hospital during the study duration without DMT2 were requested to take part in the study as controls. Brief history, including the duration of diabetes mellitus, symptoms, medication, etc. was documented. In patients with DMT2, blood samples were drawn at 9:00 AM to measure the vitamin D levels. Vitamin D deficiency was declared if the levels were lower than 30 ng/ml and sufficient if the levels were equal to or greater than 30 ng/ml. All this information was collected through a pro forma.

All the collected information was analyzed using Statistical Package for the Social Sciences, SPSS version 26 (IBM Corp., Armonk, NY). The continuous study variables like age and duration of DMT2 were presented as mean with standard deviation. For categorical parameters like gender, age groups, the severity of the deficiency, frequency, and percentages were deduced. For finding the association between vitamin D deficiency and DMT2, the Chi-square test was applied. A p-value of < 0.05 was set as a statistical cut-off for significance. 

## Results

The mean age in patients with diabetes mellitus type 2 (DMT2) was 50 ± 5.5 years. The majority of the participants were older than 45 years. The males were predominant in our study. The mean duration of DMT2 was 6.8 ± 2.4 years. There was no significant difference between patient characteristics of case and control groups (Table 1). 

**Table 1:** The Socio-demographic and Clinical Profile of Case versus Control Group

Patient Characteristics	Case	Control	p-value
Mean Age (years)	50.0 ± 5.5	52.1 ± 5.7	0.755
40-45 years	136 (25.9%)	143 (27.2%)	0.906
46-50 years	148 (28.2%)	138 (26.3%)	
51-55 years	148 (28.2%)	151 (28.8%)	
56-60 years	94 (17.9%)	93 (17.7%)	
Gender			
Men	284 (54.1%)	280 (53.3%)	0.805
Women	241 (45.9%)	245 (46.7%)	
Mean Duration of Diabetes Mellitus Type 2 (years)	6.8 ± 2.4	-	
1-3 years	77 (14.7%)	-	
4-6 years	136 (25.9%)	-	
7-9 years	270 (51.4%)	-	
10 years	43 (8.2%)	-	

The mean vitamin D levels in the case and control groups were 22.3 ± 10.4 ng/ml and 28.6 ± 12.8 ng/ml, respectively. Individuals with DMT2 were more likely to suffer from severe vitamin D deficiency (p<0.0001). The rate of severe vitamin D deficiency in the case group was 12.4% versus 4.8% in the control group. Vitamin D deficiency was significantly associated with the occurrence of DMT2 (p<0.0001) (Table 2). 

**Table 2:** The Association of Vitamin D deficiency and Occurrence of Diabetes Mellitus Type 2

Parameter	Case	Control	p-value
Severity of Vitamin D Deficiency			
Severe Deficiency (1-10 ng/ml)	65 (12.4%)	25 (4.8%)	<0.0001
Deficiency (11-20 ng/ml)	218 (41.5%)	125 (23.8%)	
Suboptimal Levels (21-30 ng/ml)	153 (29.1%)	185 (35.2%)	
Upper Normal Levels (31-40 ng/ml)	88 (16.8%)	190 (36.2%)	
Vitamin D Level Categories			
Normal	241 (25.9%)	375 (71.4%)	<0.0001
Vitamin D Deficiency	284 (54.1%)	150 (28.6%)	

## Discussion

According to the existing literature, the resistance of insulin and beta-cell dysfunction is linked with low levels of vitamin D and an increase in HbA1C levels. Metabolic syndromes are associated with deficiency of vitamin D which in turn led to the high resistance of insulin and decreased production of insulin [11]. The present study aimed to evaluate the association between diabetes mellitus type 2 (DMT2) and vitamin D deficiency.

Literature suggests that replenishing serum vitamin D may help to improve secretion of insulin and treat glycemia in DMT2 patients with concomitant vitamin D deficiency. Previous research has shown that the majority of patients with DMT2 had vitamin D deficiency when compared to patients with type 1 diabetes [12-14]. This research proved that deficiency of vitamin D is more likely to occur in patients with DMT2 as compared to patients with type 1. Hidayat et al. found a link between DMT2 and vitamin D deficiency by reporting a high rate of vitamin D deficiency of 78% [13]. In our study, we found that 54.1% of DMT2 patients had inadequate levels of DMT2. 

Furthermore, Qu et al. in their study analysis the link between vitamin D deficiency and diabetic peripheral neuropathy in patients with DMT2 [15]. Deficiency of vitamin D was linked to the development of diabetic peripheral neuropathy in Caucasian patients in their study. Asian diabetic patients who had vitamin D deficiency were more likely to suffer from diabetic peripheral neuropathy as compared to Asian patients who had normal vitamin D levels. The authors also advised adding vitamin D supplements in the treatment process to prevent diabetic peripheral neuropathy in patients with DMT2. Similarly, Luo et al. found similar results in their meta-analysis in which patients who presented with both DMT2 and deficiency of vitamin D also had an increased risk of diabetic retinopathy [16].

The idea of prevention of DMT2 via vitamin D supplementation emerged during the past two decades [17]. A large longitudinal cohort study on the Danish population revealed that DMT2 risk increased considerably with a decrease in plasma vitamin D with hazard ratios of 1.22 (95% CI; {0.85-1.74}) for vitamin D levels between 12 and 50 nmol/L [18]. In a study by Jumaa and colleagues, it was revealed that the levels of vitamin D were significantly lower in patients with DMT2 as compared to non-diabetics [19]. Furthermore, the study also revealed that young patients with DMT2 had significantly lower levels of vitamin D levels as compared to DMT2 patients aged over 18 years. Sacerdote et al. reviewed the literature extensively to find evidence indicating a correlation between vitamin D deficiency and DMT2. The study reported that current evidence suggests that there is an association between DMT2 and insulin disorders with vitamin D status; however, further studies are warranted [20]. 

Few other studies revealed a connection between lower all-cause mortality and high vitamin D levels. A study by Wan et al. concluded that high plasma vitamin D levels were significantly correlated with lower overall mortality among individuals with DMT2 [21]. Similarly, another study by Lu et al. revealed that among 15195 adults only 23% had sufficient levels of vitamin D and patients with vitamin D levels of below 30 nmol/L had significantly higher all-cause mortality (p<0.001) [22]. Therefore, by ensuring that patients do not suffer from low levels of vitamin D, the mortality rate can be reduced.

In conclusion, current findings and existing evidence suggest that vitamin D may help in preventing or at the most delaying the onset of DMT2. Our study was limited by a small and undiversified sample size which prevents us from applying the findings of the current study to a larger Pakistani population. Further, large-scale, multi-culture studies are warranted. 

## Conclusions

The current study indicates that serum vitamin D is inversely associated with diabetes mellitus type 2 (DMT2). The study revealed that the frequency of vitamin D deficiency in patients with DMT2 was 83.2% which is considerably high. In short, vitamin D deficiency seems to be involved in the pathogenesis of DMT2, and supplementation of vitamin D can aid in the prevention of DMT2. However, note that this is only a conjecture; large-scale studies are warranted in order to ascertain these claims. Nevertheless, generally, physicians should always consider vitamin D deficiency in patients with DMT2. 
